# Formation of Polymer Walls through the Phase Separation of a Liquid Crystal Mixture Induced by a Spatial Elastic Energy Difference

**DOI:** 10.1038/s41598-019-46810-8

**Published:** 2019-07-16

**Authors:** Tae-Hoon Choi, Su-Min Do, Byoung-Gyu Jeon, Sung Tae Shin, Tae-Hoon Yoon

**Affiliations:** 1 Department of Electronics Engineering, Pusan National University, Busan, 46241 Korea; 2 Department of Display and Semiconductor Physics, Korea University, Sejong, 30019 Korea

**Keywords:** Displays, Applied physics, Electrical and electronic engineering

## Abstract

We propose a method to form polymer walls without the use of a photomask in a liquid crystal (LC) cell by phase separation of an LC mixture induced by a spatial elastic energy difference. When an in-plane electric field is applied to a vertically aligned cell filled with a mixture of LC and a reactive monomer (RM), a high spatial elastic energy is induced along the direction perpendicular to the interdigitated electrodes. RMs move to the boundaries where the elastic energy is very high and an in-plane component of the applied electric field exists, which results in the phase separation of the LC/RM mixture. We have shown that we can form polymer walls by applying ultraviolet light irradiation to the LC cell. These polymer walls can function as alignment layers. We observed morphological patterns of the polymer structure through polarized optical microscopy, scanning electron microscopy, and atomic force microscopy. The polymer walls formed in an LC cell can affect the orientation of LCs in the lateral direction. Bistable switching of a polymer-walled cell could be achieved by using three-terminal electrodes where both vertical and in-plane electric fields can be applied. Vertical anchoring with the alignment layer on each substrate allows LC molecules to remain vertically aligned after removal of the applied vertical electric field. Furthermore, in-plane anchoring with the formed polymer walls allows the LC molecules to remain homogeneously aligned after removal of the applied in-plane electric field. The proposed method for the formation of polymer structures could be a useful tool to fabricate LC cells for various applications. As a bistable phase-grating device, the diffraction efficiency of a polymer-walled cell was comparable to that of a pure-LC cell. Its operating voltage was 44% lower than that of a pure-LC cell owing to in-plane anchoring provided by the polymer walls. Moreover, it can be operated with very low power because it does not require power to maintain the state. In addition, the total response time of a polymer-walled cell was approximately 68% shorter than that of a pure-LC cell because all switching was forcibly controlled by applying an electric field.

## Introduction

Liquid crystals (LCs), a type of material whose optical characteristics can be controlled by applying an electric field, have been widely used in various optical and photonic devices as well as display devices. LC/polymer composites can further broaden the range of applications because polymers act as multifunctional materials. A polymer structure built inside an LC cell can change properties such as the alignment, orientational ordering, and anchoring energy of LC molecules through physical or chemical interactions between the LC and polymer^1–7^. Photopolymerization is widely used over thermal curing because of the advantages of a high rate of polymerization and environmental benefits resulting from the elimination of organic solvents. In such photopolymerized systems, the formation of the polymer structure is based on photoinduced phase separation in a mixture of a nonreactive LC and a reactive monomer (RM). The physical mechanism of the phase separation of an LC/RM mixture has been investigated through several theoretical and experimental approaches^8–16^. One is the phase separation of an LC/RM mixture resulting from the spatial difference in the intensity of the electric field^8–10^. A large gradient in the electric field induces phase separation between LCs and RMs because both LCs and RMs are dielectric materials, but they have different dielectric constants. According to Kelvin theory, LC molecules are forced to move toward higher-electric-field regions while the RM is pushed to lower-electric-field regions because the employed LCs typically have a higher dielectric constant than the RM. Another mechanism of the phase separation of an LC/RM mixture is based on a spatial elastic energy difference^11–16^. The topological defects or associated distortions of the LC molecules induce a large spatial elastic energy difference. RM tends to phase separate toward domain boundaries where the elastic energy of the host LC distortion is highest to minimize the free energy of the overall composite.

Recently, our group reported the effect of two-dimensional (2D) confinement on switching of nematic LCs by an in-plane electric field^17–22^. When an electric field is applied to an LC cell with interdigitated electrodes, LC molecules are reoriented in the opposite direction at the edges of the interdigitated electrodes, and virtual walls are built at the center of the interdigitated electrodes and in the gaps between them. At the domain boundaries, there is no change in the azimuth or polar angle of the LC director, and therefore, the LC molecules are two-dimensionally confined not only by the two substrates but also by the domain boundaries, which are treated as virtual walls^17–22^. In these 2D-confined cells, the LC response time is dependent on the distance between the virtual walls in the lateral direction as well as the cell gap between the two substrates in the longitudinal direction, as a result of anchoring provided by the virtual walls. The 2D-confined LC cells exhibited a very short response time, which can be an outstanding feature for such applications as augmented/virtual reality, phase grating, and window display devices^17–26^. In addition, the deformation of 2D-confined LCs generates interesting physical and optical phenomena in the LC cell. When an electric field is applied to the LC cell, a large spatial elastic energy difference is induced along the direction perpendicular to the interdigitated electrodes resulting from 2D confinement with virtual walls. Electric-field-induced spatial elastic energy difference enables the formation of fine polymer walls because the spacing between the polymer walls can be controlled simply by the pitch of the interdigitated electrodes. Moreover, the polymer structure that is templated by the periodically deformed host LC may impose an orientation force in the lateral direction. The formation of polymer walls can be a very attractive method to fabricate a flexible LC cell using plastic substrates. A polymer-walled cell is free from image quality degradation caused by the LC flow and variation in the cell gap in a flexible device because it can provide mechanical stability to inhibit bending of the LC cell. The polymer walls can be built by irradiation of ultraviolet (UV) light through a photomask^27–30^, but this method may not be suitable for constructing a fine polymer structure.

In this work, we propose a method to form polymer walls without the use of a photomask through phase separation of an LC/RM mixture induced by a spatial elastic energy difference. When an in-plane electric field is applied to a vertical alignment (VA) cell containing an LC/RM mixture, a large spatial elastic energy is induced along the direction perpendicular to the interdigitated electrodes. The RMs move to the center of interdigitated electrodes and to the middle of the gaps between them, where the elastic energy is very high and no in-plane component exists in an applied electric field. We can form polymer walls by irradiating the LC/RM mixture with UV light. The polymer walls can contribute to the orientation of LCs in the lateral direction. For bistable switching of a polymer-walled cell, we used a three-terminal electrode structure where both vertical and in-plane electric fields can be applied. We have shown that with the fabricated polymer-walled LC device, 93.2% of the incident light could be transferred from the zeroth order to higher orders, and a 2^nd^-order diffraction efficiency of 21.8% could be achieved.

## Results and Discussion

### Elastic energy distribution in an LC cell

**Figure 1:**
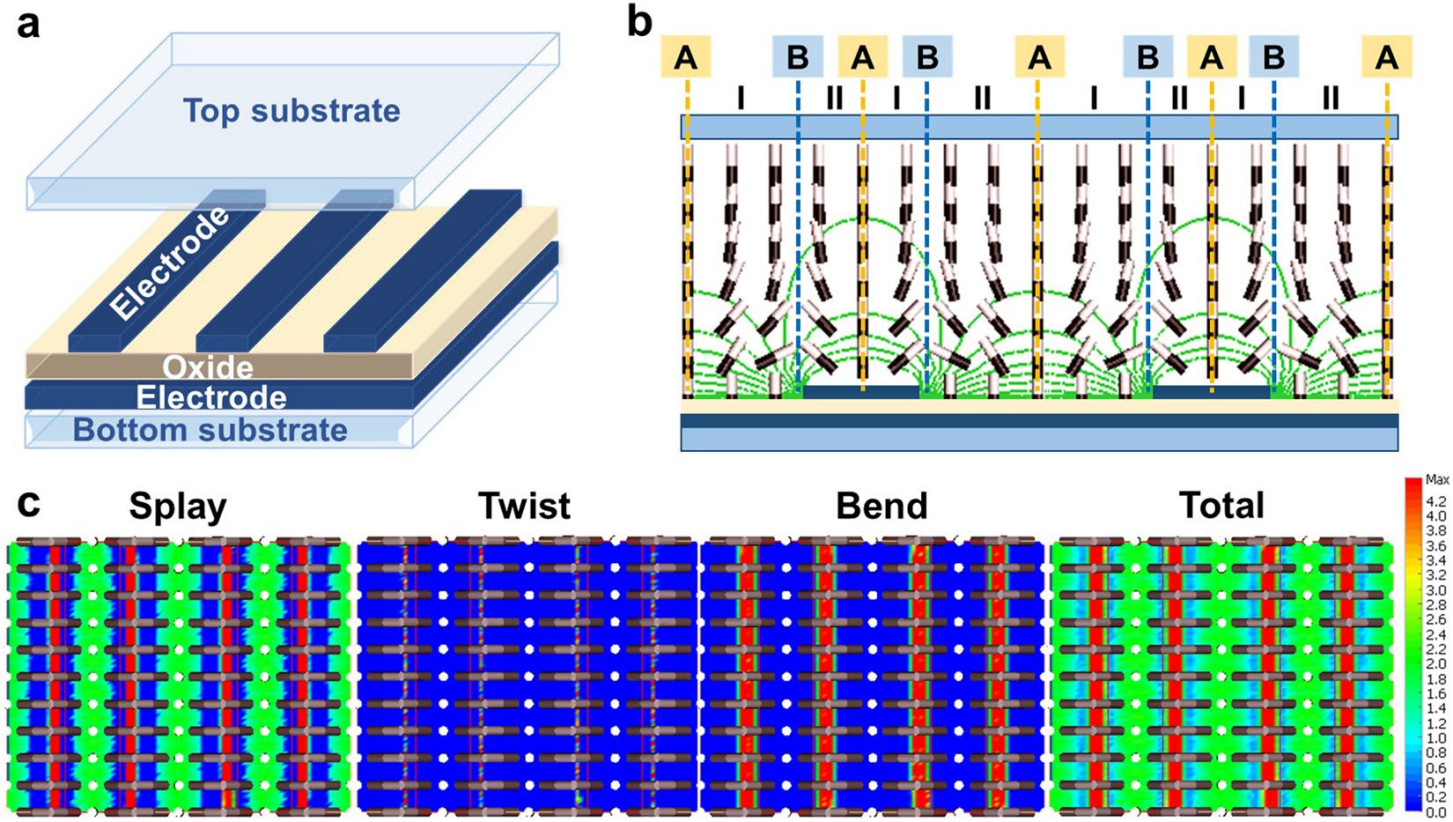
(**a**) Structure of an LC cell with double-layered electrodes. (**b**) LC director orientation and equipotential lines in a VA cell driven by an in-plane electric field. (**c**) Splay, twist, bend, and total elastic energy profiles in a VA cell driven by an in-plane electric field.

In this study, we employed a VA cell with double-layered electrodes among 2D-confined LC cells because of its easy fabrication, which does not require a rubbing process. In the double-layered electrodes, a planar electrode with no pattern was separated from interdigitated electrodes with an oxide dielectric layer on the bottom substrate, whereas no electrode was deposited onto the top substrate, as shown in Fig. 1(a). Initially, LCs with positive dielectric anisotropy were vertically aligned so that there was no spatial elastic energy difference in the LC cell. When an in-plane electric field was applied to the LC cell, the LC molecules in region I are tilted down in the clockwise direction, whereas those in region II are tilted down in the counter-clockwise direction. At the A boundaries, there are no in-plane components of the applied electric field, as shown in Fig. 1(b), and there is no change in the polar angle of the LC director owing to the symmetric LC distribution around the boundaries. However, at the B boundaries near the edges of the interdigitated electrodes, the in-plane component of the applied electric field is very high, and the electric field distribution is not symmetrical. The LC molecules on the right of these boundaries are rotated in the opposite direction to those on the left because the LC molecules with a positive dielectric anisotropy are reoriented parallel to an applied electric field. At the B boundaries, most of the LCs remain vertically aligned, although some of the LCs at the boundaries were rotated because of the nonsymmetric electric field distribution.

Here, we consider the elastic energy density due to the redistribution of the LC molecules induced by the applied electric field. The elastic energy density of deformed LCs can be written as

1 $$E=\frac{1}{2}{K}_{11}{(\nabla \cdot \overrightarrow{n})}^{2}+\frac{1}{2}{K}_{22}{(\overrightarrow{n}\cdot \nabla \times \overrightarrow{n})}^{2}+\frac{1}{2}{K}_{33}{(\overrightarrow{n}\times \nabla \times \overrightarrow{n})}^{2},$$

where *K*_11_, *K*_22_, and *K*_33_ are the splay, twist, and bend elastic constants, respectively, and $\overrightarrow{n}$ is a unit vector representing the LC director distribution in the cell. Due to positive dielectric anisotropy, vertically aligned LC molecules can be reoriented in the xz plane under an applied electric field. In this case, let the LC director $\overrightarrow{n}$ be written as a row vector in the xyz coordinate: $\overrightarrow{n}=(0,\,\sin \,\theta ,\,\cos \,\theta )$, where *θ* is the tilt angle. In general, the tilt angle *θ* is a function of z because the LC molecules are one-dimensionally confined between the two substrates as boundaries. Furthermore, in a 2D-confined cell, the LC molecules are anchored by virtual walls as well as by the surface of the substrates so that the tilt angle *θ* is a function of both x and z. Considering this boundary condition, the divergence and curl of the direction $\overrightarrow{n}$ can be written, respectively, as

2 $$\nabla \cdot n=\,\cos \,\theta \frac{d\theta }{dx}-\,\sin \,\theta \frac{d\theta }{dz},$$

3 $$\nabla \times n=(-\,\sin \,\theta \frac{d\theta }{dx}-\,\cos \,\theta \frac{d\theta }{dz},0,0).$$

Substituting Eqs 2, 3 into Eq. 1, we can obtain the elastic energy density. For a 2D-confined VA cell with a small distortion (*θ* ≈ π/2), the elastic energy density can be written as

4 $$E=\frac{1}{2}{K}_{11}{(\frac{d\theta }{dx})}^{2}+\frac{1}{2}{K}_{33}{(\frac{d\theta }{dz})}^{2}.$$

Elastic energy is associated with the square of the spatial variation of tilt along the x-axis as well as that along the z-axis. The elastic energy density consists of the sum of the splay elastic energy density and the bend energy density. In other words, both bend and splay deformation occur in an LC cell when an electric field is applied between the interdigitated and common electrodes.

To investigate the elastic energy profiles in an LC cell, the Ericksen–Leslie equation, coupled with the Laplace equation, was solved numerically using the finite-element method. The Ericksen–Leslie equation is generally used to describe the motion of the LC director. Numerical calculations were performed using a commercial software package TechWiz LCD 2D (Sanayi System Company, Ltd., Korea). Figure 1(c) shows the calculated LC director distribution and the resultant elastic energy profiles under an applied in-plane electric field. As described above, splay deformation and bend deformation occur, but there is no twist deformation. The field-induced splay deformation contributes to the induction of a large spatial total elastic energy difference in the horizontal (x-axis) direction, as shown in Fig. 1(c). The total elastic energy is highest at both boundaries A and B, as shown in Fig. 1(c). The in-plane component of the applied electric field is very high at the B boundaries, whereas there are no in-plane components of the applied electric field at the A boundaries, as shown in Fig. 1(b). According to the previously reported mechanisms of the phase separation^8–16^, RMs are expected to drift to the A boundaries if an LC/RM mixture is injected into the cell and an electric field is applied to the cell. With the help of the phase separation induced by a spatial elastic energy difference, we could form polymer walls at the A boundaries of the cell through the UV curing process without the use of photomasks.

### Phase separation in an LC/RM mixture

**Figure 2:**
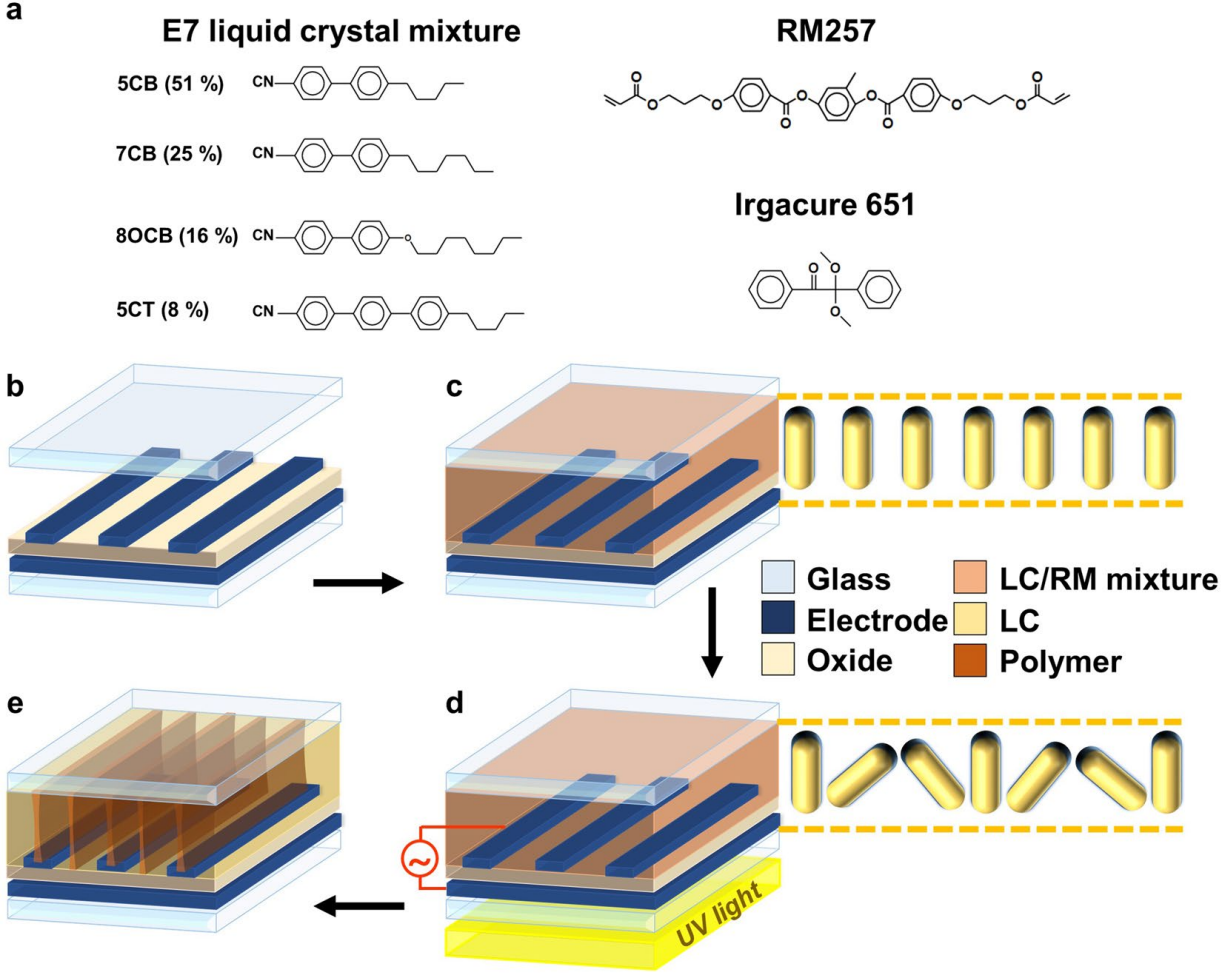
(**a**) Chemical structures of the used materials. Photopolymerization process for the fabrication of an LC cell with polymer walls: (**b**) preparation of an empty cell with double-layered electrodes, (**c**) injection of an LC/RM mixture into an empty cell, (**d**) UV exposure for polymerization under an applied in-plane electric field, and (**e**) completion of an LC cell with polymer walls.

To confirm the phase separation in an LC/RM mixture due to a spatial elastic energy difference, an LC cell was fabricated. We prepared an LC/RM mixture, and the chemical structures of the materials used are shown in Fig. 2(a). We used Merck E7 as the host LC, whose material parameters are as follows: dielectric anisotropy ∆ε = 13.8 (ε_∥_ = 19, ε_⊥_ = 5.2); optical anisotropy ∆n = 0.2253 (n_e_ = 1.746, n_o_ = 1.522 at 589 nm, 20 °C); elastic constants *K*_11_ = 11.1 pN, *K*_22_ = 10.3 pN, *K*_33_ = 17.1 pN; and rotational viscosity γ_1_ = 250 mPa·s. UV-curable monomer RM257 containing a small amount of the photoinitiator Irgacure 651 was used in our experiment. RM257 has a rod-like structure and can be easily aligned with LCs. The LC/RM mixture was composed of 97.0% host LC and 3.0% UV-curable monomer. The LC/RM mixture was stirred for 12 h, after which an ultrasonic wave was applied for 1 h. The overall fabrication process to form polymer walls is shown in Fig. 2(b,d). The width of the interdigitated electrodes placed on the bottom substrate and the distance between them were 2.8 μm and 6 μm, respectively. We coated a vertical alignment layer on each substrate, which was baked for 1 h at 230 °C. The cell was assembled using silica spacers with diameters of 10 μm, as shown in Fig. 2(b). Next, the prepared LC/RM mixture was injected into an empty cell via capillary action, as shown in Fig. 2(c). Initially, the LC/RM mixture was vertically aligned by the alignment layer on each substrate so that there was no spatial elastic energy difference in the cell. When an electric field was applied between the interdigitated and common electrodes, as shown in Fig. 2(d), the LC/RM mixture switched, and both splay and bend deformation occurred, as described in the above section, so that a large spatial elastic energy difference was induced along the direction perpendicular to the interdigitated electrodes. The RMs moved to the A boundaries of Fig. 1(b), where there is no in-plane component of the applied electric field and the elastic energy is very high, which resulted in phase separation of the LC/RM mixture. Under the applied electric field, the LC cell was exposed to the spatially uniform UV light with a weak intensity of 2 mW/cm^2^ to polymerize the RM, as shown in Fig. 2(d). Polymer walls were built through curing the RM under an applied electric field, as shown in Fig. 2(e). Finally, polymer walls were built at the center of the interdigitated electrodes and in the middle of the gaps between them, which correspond to the A boundaries in the LC cell shown in Fig. 1(b).

**Figure 3:**
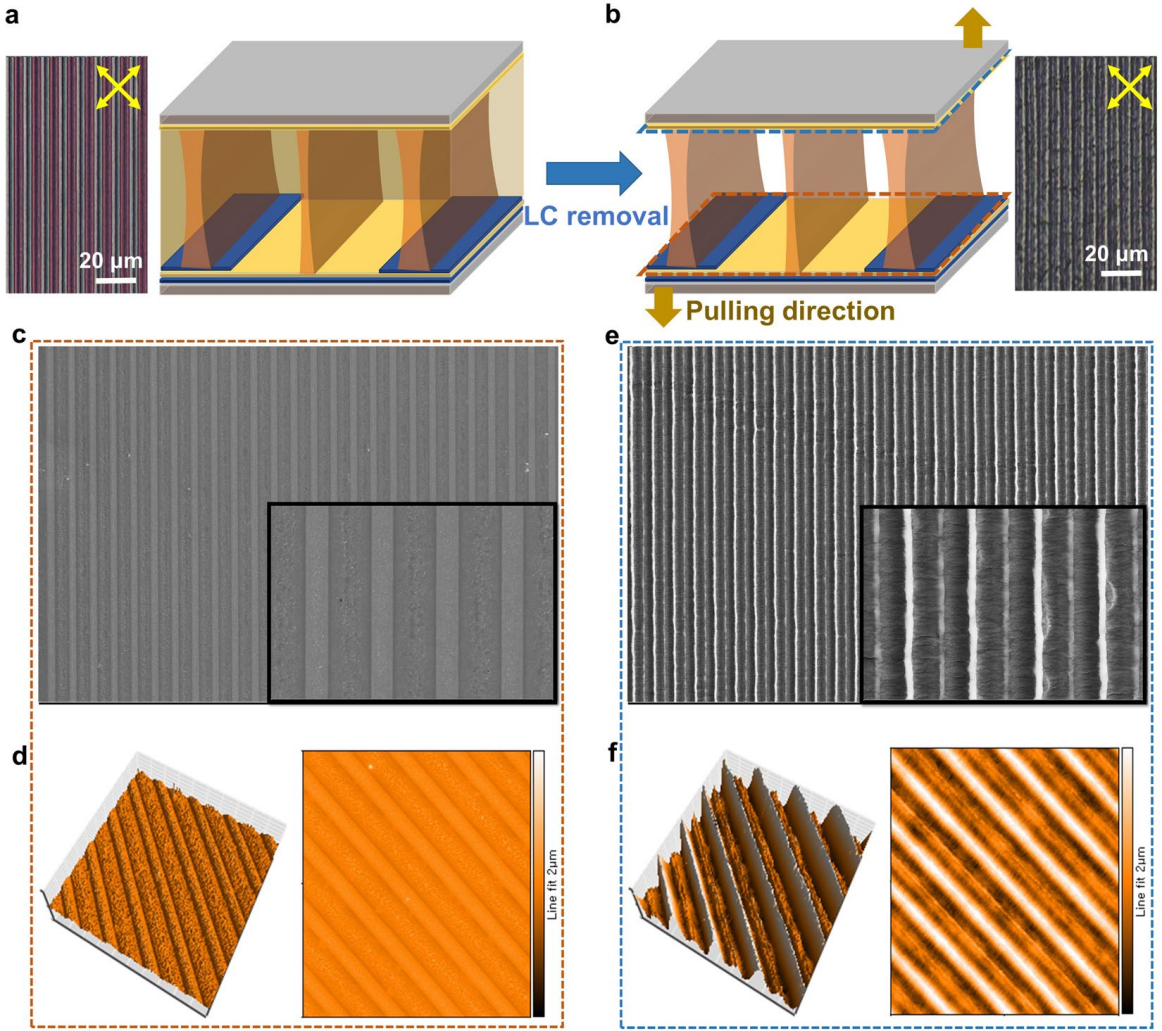
POM images and cell structures (**a**) before and (**b**) after removal of LCs. (**c**) SEM and (**d**) AFM images of the separated bottom substrate. (**e**) SEM and (**f**) AFM images of the separated top substrate. The insets in (**c**) and (**e**) present magnified views of the SEM images.

To identify the formation of polymer walls, we observed the fabricated LC cell using polarized optical microscopy (POM), scanning electron microscopy (SEM), and atomic force microscopy (AFM), as shown in Fig. 3. The periodic deformation of LCs was induced by applying an electric field, and the LCs were used as the reaction template. The polymerization of RMs by UV light under an applied electric field resulted in a morphologically patterned polymer structure whose chain orientation was dependent on the orientation of the LC molecules. Consequently, the overall optical texture retained its pattern with removal of the electric field, as shown in Fig. 3(a), although slight relaxation of the local director was observed. To indirectly confirm the polymer structure built within the cell, we also recorded POM images of the cell after the removal of LC, as shown in Fig. 3(b). Nonreactive LCs were removed with an organic solvent, while the polymer structure remained in the cell. The cell shows brightness patterns between crossed polarizers because of the weak birefringence of the dense bundles of the polymerized RMs, where the position of the bright lines corresponds to the center of interdigitated electrodes and the middle of the gaps between them.

To confirm the polymer structure in more detail, we carefully separated the two substrates of the cell after LC removal, as shown in Fig. 3(b). We then observed SEM and AFM images of each separated substrate, as shown in Fig. 3(c,f). Figure 3(c,d) show SEM and AFM images of the separated bottom substrate with the interdigitated electrodes, respectively. The SEM image exhibits a continuous polymer structure with periodic polymer walls, and a uniform morphological pattern was observed throughout the entire active area of the bottom substrate, as shown in Fig. 3(c). These periodic polymer walls could also be confirmed by employing AFM analysis, as shown in Fig. 3(d). The surface profile of the bottom substrate showed periodic microgrooves in the direction perpendicular to the interdigitated electrodes, which were generated by the formed polymer structure. The distance between the highest regions of the root-mean-square roughness corresponds to the distance between the A boundaries of Fig. 1(c). These POM and SEM results verify that the polymer structure was suitably located at the center of the interdigitated electrodes and the middle of the gaps between them by phase separation due to a spatial elastic energy difference. The polymer structure was also observed on the top substrate, as shown in Fig. 3(e,f). The polymer structure of the top substrate was thicker than that of the bottom substrate, which indicates that polymer bundles far from the bottom substrate would be thicker.

As explained in the above section, when an electric field is applied to a cell containing an LC/RM mixture, phase separation occurs due to the induced spatial elastic energy difference so that RMs move to the A boundaries where there is no in-plane component of the applied electric field. Although there is no in-plane component of the applied electric field at the A boundaries, a weak vertical component still exists, as shown in Fig. 1(b). At these boundaries, the vertical component of the applied electric field has a gradient in the direction perpendicular to the substrate because an electric field is applied between the interdigitated and common electrodes on the bottom substrate. Therefore, because the position is closer to the top substrate, the intensity of the vertical component of the applied electric field is weaker, which may result in a denser polymer structure near the top substrate. In other words, relatively smooth-shaped polymer walls were built in a direction perpendicular to the substrate when compared to those fabricated with a photomask. In addition, a polymer structure with different shapes was observed at the center of the interdigitated electrodes and the middle of the gaps between them, as shown in Fig. 3(c,e). This effect is closely related to the director configuration and the spatial elastic energy distribution, which is dependent on the width of the interdigitated electrodes and the gap between them. In our experiments, the width of the interdigitated electrodes was approximately half the length of the gap between them. The elastic energy density at the center of the interdigitated electrodes was higher than that at the middle of the gaps between them because the LC molecules near the boundaries at the center of the interdigitated electrodes are more deformed. This resulted in the formation of a thicker polymer structure at the center of the interdigitated electrodes than that in the middle of the gaps between them.

**Figure 4:**
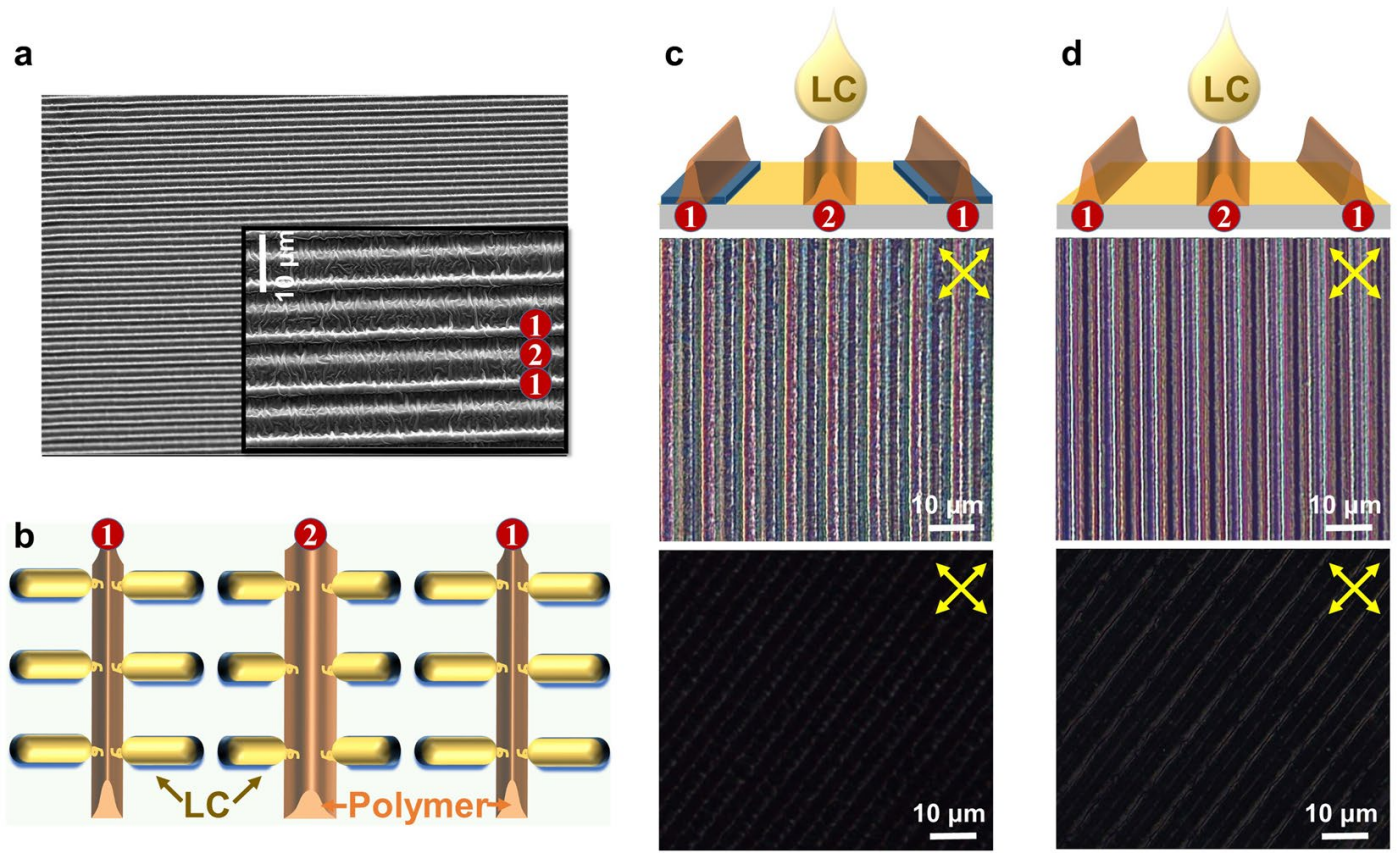
(**a**) SEM images of the polymer structure formed on the top substrate and (**b**) a schematic representation of the orientation of LC molecules induced by anchoring with the polymer structure. POM images observed after the top and bottom substrates were separated and LCs were dropped and spin-coated on the (**c**) bottom and (**d**) top substrates.

These polymer walls formed in the cell can play a role in aligning the LC molecules in the direction perpendicular to the polymer walls by anchoring between the LC and polymer structure, as shown in Fig. 4. In a polymer-walled cell, the alignment layer on each substrate forces the LC molecules to align along the longitudinal direction, whereas an alignment force to align LC molecules in the lateral direction due to the formed polymer walls also exists. The polymer walls are formed at the center (marked by ①) of the interdigitated electrodes and at the middle of the gaps between them (marked by ②), as shown in the SEM image of Fig. 4(a), which was obtained at a tilt angle of 30°. Although the shape of the polymer walls was different in ① and ② due to the electrode structure, all of their chains were aligned along the direction perpendicular to the formed polymer walls. The fibril-like morphology of the local polymer bundles can precisely replicate the local director orientation of the host LCs. Therefore, LC molecules can be homogeneously aligned in the direction perpendicular to the polymer walls, as shown in Fig. 4(b), and the alignment force of the LC molecules by the polymer structure originates from the local molecular interaction between the LC molecules and polymers.

To confirm the alignment force of the LCs by the polymer walls, the top and bottom substrates were separated, and then, LCs were dropped and spin-coated on each substrate, as shown in Fig. 4(c,d). In this case, the orientation force by the alignment layer existed only on a single substrate because each substrate was separated. At the air interface, the surface tension force may cause the LC molecules to align perpendicular to the substrate, but its alignment force is very weak compared to that of the alignment layer or polymer. When the LCs were dropped on the top and bottom substrates with the polymer structure, they were well-oriented in the direction perpendicular to the interdigitated electrodes due to the interaction between the LCs and polymer, as shown in Fig. 4(c,d). When the interdigitated electrode on the bottom substrate was set to 45° with respect to the transmission axis of one of the crossed polarizers, we observed periodic bright stripes, as shown in the top image of Fig. 4(c). However, when the direction of the interdigitated electrode was set along the transmission axis of one of the crossed polarizers, the substrate was dark, but there was a slight leakage of light due to the birefringence of the polymer structure, as shown in the bottom image of Fig. 4(c). These POM images clearly confirm that LCs are homogeneously aligned, and the alignment direction of the LC molecules coincides with the direction parallel or perpendicular to the interdigitated electrodes. Because the chains of the polymer walls are aligned along the LC orientation direction during polymerization, the LC molecules are aligned in the direction perpendicular to the interdigitated electrodes. These results were also observed on the top substrate where there is no interdigitated electrode, as shown in Fig. 4(d). In other words, the polymer walls formed in the cell function as alignment layers so that the LC molecules are homogeneously aligned in the direction perpendicular to the polymer walls.

### Switching of a polymer-walled cell

**Figure 5:**
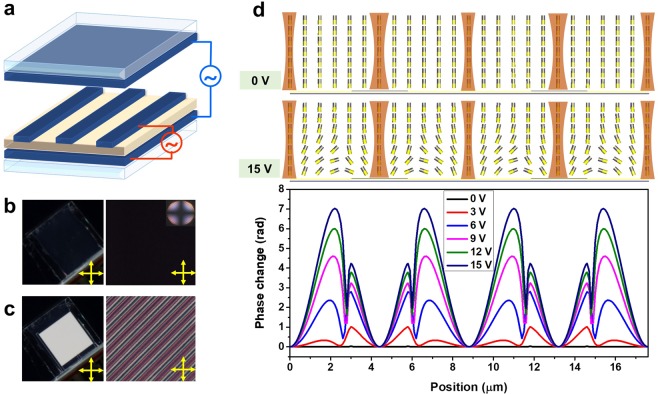
(**a**) Three-terminal electrodes by which both vertical and in-plane electric fields can be applied. Photographs of a cell placed between crossed polarizers and its POM images after removal of the (**b**) vertical and (**c**) in-plane electric field. (**d**) Calculated LC director distributions and phase difference profiles in a VA cell driven by an in-plane electric field with the applied voltage as a parameter.

So far, we have confirmed that polymer walls could be formed in an LC cell by phase separation of an LC/RM mixture, and this polymer structure could contribute to the orientation of LCs in the lateral direction. For bistable switching of the device based on the existence of the orientation force in both the longitudinal and lateral directions, we used three-terminal electrodes by which both vertical and in-plane electric fields can be applied, as shown in Fig. 5(a). An in-plane electric field can be applied between interdigitated and common electrodes on the bottom substrate, whereas a vertical electric field can be applied between the top and bottom electrodes. We fabricated a polymer-walled LC cell with parameters and a fabrication process that were the same as those explained in the previous section except for the presence of a top electrode.

To confirm the bistable switching ability between vertically and homogeneously aligned states, we observed the fabricated cell placed between crossed polarizers after removing the electric field applied to the cell, as shown in Fig. 5(b,c). When a vertical electric field is applied to a polymer-walled cell, a strong dielectric torque pulls the LC molecules to the vertical direction so that LC molecules are vertically aligned. In this case, vertical anchoring due to the alignment layer on each substrate allows the LC molecules to remain vertically aligned after removal of the applied vertical electric field. The LC molecules retain the vertical alignment so that the cell shows a dark state between crossed polarizers, as shown in Fig. 5(b). The conoscopic figure in the inset of Fig. 5(b) confirms the vertical orientation of the anisotropic LC/RM mixture after removal of the applied vertical electric field. When an in-plane electric field is applied to the cell, LCs with a positive dielectric anisotropy are reoriented along the direction of the applied electric field. In addition to the electric-field-induced dielectric torque, an orientation force exists along the horizontal direction due to the formation of polymer walls in the cell. The polymer walls were built from mesogenic monomers with flexible tails and flexible monomers. The polymer walls are fiber-like and anisotropic, and their chains are aligned along the LC orientation direction during polymerization. The polymer chains are oriented in the direction perpendicular to the interdigitated electrodes because the polymer walls formed through polymerization under an applied in-plane electric field, which resulted in the homogeneous alignment of LC molecules after removal of the applied in-plane electric field by the interaction between LC molecules and polymer surfaces. Here, the anisotropic-shaped monomers similar to the LC molecules would contribute to imposing strong anchoring between the LC and polymer structure. Thus, after removal of the applied in-plane electric field, the cell placed between the crossed polarizers showed a bright state and periodic bright stripes were observed in the POM image, as shown in Fig. 5(c). In other words, bistable switching between the vertical and homogeneous aligned states could be achieved via orientation forces existing in both the longitudinal and lateral directions. This LC cell capable of bistable switching can be used to control the polarization of light propagating through it while controlling the brightness when placed between crossed polarizers. However, the transmittance of the cell is lower than that of other LC display devices because there is little phase retardation at the A and B boundaries of Fig. 1(b). The dead zones around the boundaries correspond to periodic black lines in the POM image, as shown in Fig. 5(c).

These boundaries can decrease the transmittance, and they can induce a large spatial phase difference as an LC phase grating, as shown in Fig. 5(d). When the vertical electric field applied to a polymer-walled cell is removed, the LC molecules remain vertically aligned by the orientation force due to the alignment layer on each substrate. When the LC molecules are vertically aligned, the incident light is not diffracted because there is no spatial phase difference. Although periodic interdigitated electrodes and polymer walls can also cause diffraction, their contribution is negligibly small compared to the diffraction caused by the index modulation induced by the applied electric field. On the other hand, light scattering caused by the refractive index mismatch between the LC and polymer matrices would increase in the oblique view, which may cause the angular dependence of light transmittance and diffraction efficiency. We need to use refractive-index-matched LC and polymer materials to minimize the angular dependence of the light transmittance and diffraction efficiency. When an in-plane electric field is applied to a polymer-walled cell, the LC molecules are tilted down in the direction of the applied electric field. During this process, the LC molecules are also subjected to the orientation force due to the interfacial interaction between the LC and polymer. After removing the applied in-plane electric field, the LC molecules remain homogeneously aligned, which contributes to the continuous periodic LC profile. Therefore, a large spatial phase difference is induced along the direction perpendicular to the interdigitated electrodes due to a change in the refractive index of the LC layer causing the incident light to be strongly diffracted.

**Figure 6:**
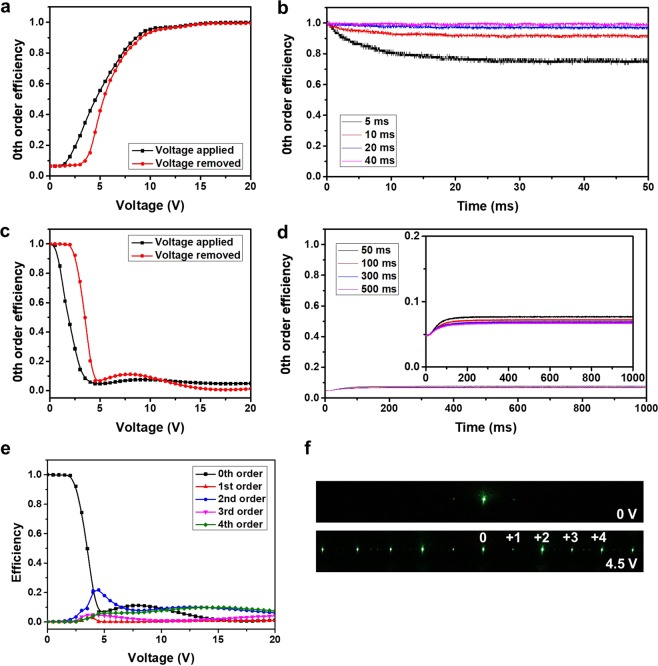
(**a**) The measured zeroth-order diffraction efficiency vs. the applied voltage while a vertical electric field is applied and after the applied field is removed. (**b**) Dependence of the temporal switching behavior on the voltage pulse duration after the applied vertical electric field is removed. (**c**) The measured diffraction efficiency of the zeroth order while an in-plane electric field is applied and after the applied field is removed. (**d**) Dependence of the temporal switching behavior on the voltage pulse duration after an applied in-plane electric field is removed. (**e**) The measured diffraction efficiencies for each diffraction order vs. the applied voltage after removal of the applied in-plane electric field. (**f**) Diffraction patterns of the fabricated polymer-walled cell.

To verify the diffraction characteristics of the fabricated cell, we measured the diffraction efficiency of the zeroth order both while an electric field was applied and after the applied field was removed. We illuminated the LC cell using a linearly polarized He-Ne laser beam (wavelength λ = 543.5 nm). The incident laser beam was polarized in the direction perpendicular to the interdigitated electrodes. We measured the far-field intensity of the zeroth order using a photodiode placed 22.5 cm away from the LC cell. Figure 6(a) shows the measured diffraction efficiency of the zeroth order with an applied vertical voltage and after the applied voltage was removed. When a vertical electric field was applied, the efficiency of the zeroth order increased as the applied voltage increased, as shown in Fig. 6(a). The vertical electric field applied to the cell caused the LC molecules to vertically align so that the spatial phase difference was reduced, which resulted in a decrease in the diffracted light intensity. When the applied vertical voltage was removed, the vertically aligned LC molecules tended to remain vertically oriented by vertical anchoring owing to the alignment layer on each substrate. Although there was a slight change in the LC orientation when the applied vertical voltage was removed, there was little difference in the efficiency of the zeroth order at a high voltage. The difference in efficiency of the zeroth order between the time at which a vertical voltage was applied and after the applied voltage was removed is dependent on the duration of the applied voltage, as shown in Fig. 6(b). When a voltage wave whose duration was not sufficiently long was applied to the cell, the intensity of the zeroth order was lowered because a fraction of the LCs did not remain vertically oriented and relaxed. When the duration was longer than 40 ms, there was little change in the efficiency of the zeroth order after the applied vertical voltage was removed, as shown in Fig. 6(b).

When an in-plane electric field is applied to a vertically aligned LC cell, a spatial phase difference is induced causing the incident light to be strongly diffracted, which resulted in a decrease in the light intensity of the zeroth order, as shown in Fig. 6(c). Here, the same vertical electric field was always applied before an in-plane electric field was applied for switching to the diffracted state. Similar to the case when a vertical electric field was applied, there was a slight change in the light intensity of the zeroth order when the in-plane field was removed. This may be attributed to the difference in the interaction between the LCs and the polymer due to the electric-field-induced deformation of the LCs. In addition, an applied in-plane electric field was required for a sufficiently long time to prevent changes in the optical characteristics of the cell when the applied electric field was removed, as shown in Fig. 6(d). When the pulse duration was longer than 500 ms, which is sufficient for switching the LC molecules, the light intensity of the zeroth order was almost unchanged after the removal of the applied electric field, as shown in Fig. 6(d). Figure 6(e) shows the measured diffraction efficiency for each order when the applied in-plane electric field was removed. To eliminate the effect of the duration of the applied electric field on the diffraction efficiency, all measurements were made at 5 s after the voltage was applied or removed. As shown in Fig. 6(e), as the applied voltage was increased, the incident light was diffracted from zeroth order to higher orders. The diffraction angles for the 1^st^, 2^nd^, 3^rd^, and 4^th^ orders were 8.6°, 17.2°, 25.8°, and 34.4°, respectively. At an applied voltage of 4.5 V, 93.2% of the incident light was diffracted to higher orders (1^st^, 2^nd^, 3^rd^, and 4^th^ orders). The diffraction efficiency of the zeroth order gradually increased, then decreased, and then saturated at a high applied voltage. The highest diffraction efficiency of the 2^nd^ order was 21.8% at an applied voltage of 4.5 V. The transfer of incident light to the 2^nd^ order can be identified from the diffraction patterns shown in Fig. 6(f).

For comparison, we also measured the maximum 2^nd^-order diffraction efficiency in a pure-LC cell, which was 23.0% at an applied voltage of 8 V. The diffraction efficiency of the 2^nd^ order in a polymer-walled cell is comparable to that in a pure-LC cell. The operating voltage of a polymer-walled cell was 44% lower than that of a pure-LC cell. The reduced operating voltage originated from in-plane anchoring by the polymer structure. A polymer-walled cell could be switched at a lower applied voltage because the polymer walls force the LC molecules to be tilted down. Moreover, a polymer-walled cell can be operated with a very low power because power is not required to maintain the state.

A short response time is one of the major requirements for phase-grating applications. We measured the response times of pure-LC and polymer-walled cells. We defined the turn-on time as the transient time from 90% to 10% of the zeroth order intensity and vice versa for the turn-off time. To measure the response time of a pure-LC cell, 8 V was applied between the interdigitated and common electrodes for the turn-on and was then removed after several seconds. The measured turn-on and turn-off times of the pure-LC cell were 4.57 and 4.63 ms, respectively. In the pure-LC cell, the LC molecules in region II of Fig. 1(b) are tilted down in the direction opposite those in region I. Boundaries A and B between regions I and II can be treated as virtual walls, such that the LC molecules are confined not only by the two substrates but also by the virtual walls. This confinement effect leads to a short response time despite a large cell gap of 10 μm. In contrast to a pure-LC cell, both turn-on and turn-off switching of a polymer-walled cell require an electric field. We applied a vertical electric field of 20 V to the cell for turn-off switching, whereas we applied an in-plane electric field of 4.5 V for turn-on switching. The measured turn-on and turn-off times were 2.44 and 0.51 ms, respectively. The total response time of a polymer-walled cell is approximately 68% shorter than that of a pure-LC cell. The polymer-walled cell exhibited faster turn-on switching even at a lower applied voltage because of in-plane anchoring by the polymer walls. Moreover, turn-off switching of a polymer-walled cell does not rely on the slow relaxation of LCs but is controlled by applying an electric field, which results in a very short turn-off time^31–34^. The polymer-walled cell can also be applied as a flexible device because it can provide mechanical stability due to finely formed polymer walls.

In addition to suggesting an excellent device with bistable switching capability as an example, our results provide a simple method for constructing polymer walls without the use of a photomask. The spacing between the polymer walls can be controlled simply by the pitch of the interdigitated electrodes so that much finer polymer walls than those fabricated with a photomask can be built in the cell. Polymer walls with various shapes can be constructed by employing LC cells with various types of electrode structures, which can generate a large spatial elastic energy difference. We believe that the proposed method for the formation of the polymer structures with optical birefringence could be a useful tool for processing optical and functional materials for the fabrication of flexible LC cells for various optical and photonic applications.

## Conclusion

In summary, we proposed a method to form polymer walls without the use of a photomask in an LC cell through phase separation of an LC/RM mixture induced by a spatial elastic energy difference. When an in-plane electric field is applied to a VA cell containing an LC/RM mixture, a large spatial elastic energy is induced along the direction perpendicular to the interdigitated electrodes. By exposing the cell to UV light, polymer walls that can function as alignment layers are built at the boundaries where elastic energy is very high and there is no in-plane component of the applied electric field. We observed the polymer structure through POM, SEM, and AFM. We also confirmed the LC alignment capability of the polymer structure. The proposed method for the formation of polymer structures could be a useful tool for the fabrication of LC cells for various optical and photonic applications.

Polymer walls formed in an LC cell can contribute to the orientation of LCs in the lateral direction. By using three-terminal electrodes, with which both vertical and in-plane electric fields can be applied, we can realize a bistable phase-grating device. The LC phase-grating device exhibited a 2^nd^ order diffraction efficiency of 21.8%, which is comparable to that of a pure-LC cell. Its operating voltage was 44% lower than that of a pure-LC cell because of the in-plane anchoring provided by the polymer walls. Moreover, it can be operated with very low power because power is not required to maintain the state. In addition, the total response time of a polymer-walled cell was approximately 68% shorter than that of a pure-LC cell because all the switching was forcibly controlled by applying an electric field. Faster turn-on switching could be achieved due to in-plane anchoring by polymer walls, whereas faster turn-off switching could be achieved due to the applied vertical electric field.
